# The Effects on Energy Markets of Achieving a 1.5 °C Scenario

**DOI:** 10.3390/ijerph20054341

**Published:** 2023-02-28

**Authors:** Lars Lindholt, Taoyuan Wei

**Affiliations:** 1 Research Department, Statistics Norway, 0177 Oslo, Norway; 2 CICERO Center for International Climate Research, 0349 Oslo, Norway

**Keywords:** net zero, climate change, mitigation, energy model, integrated assessment, CGE model, fossil fuel, energy transition

## Abstract

Net zero emission scenarios are aligned with the criteria for the Paris Agreement to keep global warming below 1.5 °C. By soft-linking an energy model with a macroeconomic model, we create a similar pathway to the net zero emission scenario from the International Energy Agency (IEA) to 2050 both of demand for fossil fuels and total CO_2_ emissions. Soft-linking entails that we insert endogenous variables from one model into the other model. We implement measures such as CO_2_ taxes, improved energy efficiency, more renewables in electricity production and other sectors, easier substitution between electricity and fossil fuels for final users, and drastically limiting future production of oil, gas and coal. Our conclusion is that net zero is possible by introducing very strict measures, e.g., a high rate of energy efficiency improvement, far above what has been achieved in the past. While our partial equilibrium energy model, similar to the IEA model, overlooks the potential rebound effects, i.e., more energy used by consumers due to lower prices caused by energy efficiency improvement, our macroeconomic model does capture the rebound effects and has to implement stricter supply-side measures to reduce fossil fuel use to achieve the 1.5 °C scenario.

## 1. Introduction

To keep global temperature increase below 1.5 or 2 °C compared to pre-industrial levels, the global CO_2_ emissions will need to become net zero and potentially below zero. As indicated by the Intergovernmental Panel on Climate Change (IPCC) in its special report on 1.5 °C: “From a physical science perspective, limiting human-induced global warming to a specific level requires limiting cumulative CO_2_ emissions, reaching at least net zero CO_2_ emissions, along with strong reductions in other greenhouse gas emissions” [1]. Jones et al. [2] suggest that, essentially, the global warming stops in the case of net zero CO_2_ emissions, implying that current choices can avoid the worst impacts of global warming in the future. Net zero energy systems, where residual CO_2_ emissions are offset by removals, are crucial to achieve economy-wide net zero emissions (see e.g., [3]).

The Paris Agreement from 2016 proposes an ambitious target of limiting global warming to well below 2 °C, preferably to 1.5 °C, compared to pre-industrial levels. This was strengthened to below 1.5 °C at the Glasgow meeting in 2021. There is a strand of studies using integrated models that analyze how 1.5 °C can be reached, e.g., the IPCC-scenarios presented in Masson-Delmotte et al. [1]. In 2021, a report released by the IEA [4] described a roadmap to achieve net zero emissions for the global energy sector by 2050 (NZE hereafter), which is necessary for a 1.5 °C world.

The purpose of this study is to examine how global energy markets might be affected during the transition period of achieving net zero emissions in 2050 based on two soft-linked complementary models. Soft-linking entails that we insert endogenous variables from one model into the other model. The IEA [4] presents the most-cited and well-known NZE pathway for energy sectors (we refer to this scenario as NZE IEA). Brecha et al. [5] have assessed various scenarios, including those from the IPCC reports, and find that the NZE IEA scenario is aligned best with the requirement specified in the Paris Agreement when it comes to having the likelihood to limit global warming to 1.5 °C. To the best of our knowledge, we have not seen any studies that use energy models to test whether the NZE IEA pathway is achievable and which relevant measures to apply to reach such a pathway. By soft-linking an energy model with a macroeconomic model, this study creates a similar pathway to 2050 and analyzes how such a pathway impacts the energy markets.

We introduce various mitigation measures simultaneously to achieve NZE in 2050 in our models. Among them, we consider CO_2_ taxes, improved energy efficiency, more renewables in electricity production and other sectors, easier substitution between electricity and fossil fuels for final users, and drastically limiting future production of fossil fuels. The effect of our various measures is to reduce fossil fuel consumption and corresponding emissions to around 90 per cent from the reference scenario by 2050. Even if it is possible to reach large reductions by implementing such strict measures, it would in practice be very challenging, if not impossible. By presenting the NZE scenarios simulated by our models, the paper aims to discuss the magnitude of the various measures that are necessary to obtain NZE and whether this is realistic or not.

The remainder of the article is organized as follows. In the next section, we present a brief literature review to justify our study. In Section 3, we introduce our models and describe how the models are used to simulate reference and NZE scenarios. In Section 4, we present the results of the effects on energy markets of the simulated NZE scenarios. In Section 5, we further discuss the issues related to the simulation results. We conclude in the last section.

## 2. Literature Review

A 1.5 °C scenario refers to a simulated pathway where there is at least a 50 per cent probability for the global warming to be no more than 1.5 °C in at least 2100 and possibly above 1.5 °C in some years before 2100 [1]. Such scenarios have been developed by various institutions, including BP [6], Equinor [7], the IEA [4], and Shell [8], besides the integrated assessment model (IAM) scenarios [1,9]. Among these scenarios, the NZE IEA scenario is identified to align best with the requirement specified in the Paris Agreement in terms of the likelihood to limit global warming to 1.5°C [5]. However, we know of no other model-based studies that test and verify the achievability of the NZE IEA pathway.

The NZE IEA pathway [4] illustrates a marked transformation of the energy system from fossil fuels to renewable energy, rapid improvement of energy efficiency, and wide electrification in the economy. However, the IEA energy model is a partial equilibrium model and assumes exogenous key determinants of energy demand such as gross domestic product (GDP). In addition, the NZE IEA pathway introduces considerable energy efficiency improvement to reduce energy demand, but excludes the potential rebound effects on energy consumption due to lower energy prices [10,11,12]. It is necessary to address what additional measures are needed to offset the rebound effect in a 1.5 °C world.

Various measures can contribute to reducing emissions. Considering the limited resources available for society, degrowth is proposed to reduce GHG emissions and contribute to emissions neutrality [13]. However, degrowth implies welfare losses of certain groups and is difficult to implement, which makes the other option, “decoupling”, attractive, i.e., breaking the link between economic growth and environmental pressures like GHG emissions [14]. Decoupling provides a win–win perspective to control global warming without sacrificing economic growth. As the NZE IEA scenario assumes GDP growth at an annual rate of around 3 per cent [4], all the measures adopted in the scenario can be regarded as decoupling measures. Given the considerable barriers to implement these decoupling measures, the NZE IEA scenario was described as achievable but challenging [4].

The energy market of particularly fossil fuels in such a 1.5 °C scenario may markedly differ across scenarios simulated by different models, depending on the assumptions in the models on the adopted mitigation measures [1]. Hence, the energy market in the NZE IEA scenario can differ from other models that achieve the same global level of emission reductions, and this will be tested by this study.

## 3. Methods and Materials

The two models we have soft-linked are a computable general equilibrium (CGE) model of the world economy, GRACE [15], and a partial equilibrium model of the global energy markets, FRISBEE [16]. Descriptions of both models are presented in [17]. Both models have been used to analyze issues related to climate policies (e.g., [16,18]). We introduce policy measures by changing central parameter values in each model, so that an NZE scenario is simulated to roughly follow the pathway of both demand for fossil fuels and total CO_2_ emissions from fossil fuels in the NZE IEA.

In studies that rely on CO_2_ prices as the only climate policy measure, as is often the case in global climate policy modelling, they can be interpreted as the marginal cost of abatement [19]. In practice, other measures than CO_2_ prices are necessary. Hence, we follow NZE IEA and the IPCC special report [1] to include various energy policies and accompanying measures designed to reduce emissions such as CO_2_ prices, renewable electricity production, and efficiency improvements.

By soft-linking the models, we insert endogenous variables from one model into the other model. In this study, the endogenous regional GDP and global crude oil price generated from GRACE are used as exogenous variables for FRISBEE to simulate the 1.5 °C scenario, so that FRISBEE captures the effects on GDP and global oil price of various mitigation measures introduced in GRACE. We do not harmonize any other issues for both models in order to keep the individual advantages of each model, as explained below.

Energy market models are, in many cases, better suited to identify various energy goods and the associated costs and investments, but do not address the impacts on the overall economy due to, e.g., interactions between markets of energy and those of other goods. On the other hand, CGE models are generally better suited to study overall economic impacts indicated by, e.g., GDP growth rates, market price effects, and structural change. These differences imply that the two modelling approaches can contain different instruments to achieve emission reductions. In addition, the effects of the same measures in the models may create diverging results. While we soft-link two top-down models in this study, it seems more common to soft-link a bottom-up energy system model and a CGE model [20,21].

### 3.1. Model Description

FRISBEE is a recursive, dynamic partial equilibrium model simulating the global energy markets with 2012 as the start year. Prices are in terms of 2012 USD and exchange rates are assumed constant over time. The model is sequentially solved year by year. The energy goods in the model cover coal, gas, oil, and bioenergy, and, further, electricity (and heat) generated from feedstock of either the fossil or non-fossil fuels, assisted by a sector of transformation and distribution. Global demand equals supply for each energy good. Demands for secondary energy goods in households and industry are modeled as log-linear functions of prices and income. In addition, autonomous energy efficiency improvement (AEEI) is implemented in the model. FRISBEE elaborately models the global oil market and regional gas markets while, in less detail, modelling the world markets for electricity, coal, and renewables. The oil price in the world market is exogenous as the residual demand is satisfied by OPEC as the difference between world demand and Non-OPEC supply at the prevailing price. FRISBEE also elaborately describes oil and gas investments and production, explicitly accounting for discoveries, reserves, and field development.

GRACE is a multi-sectoral, multi-regional recursive dynamic CGE model for the global economy. The model is calibrated to mimic the global economy in 2014. All economic values are stated in 2014 USD and exchange rates are assumed constant over time. Like FRISBEE, the GRACE model is sequentially solved year by year. A regional economy consists of 15 production activities including agriculture, forestry, fishery, three manufacturing sectors, services, three transport sectors, and five energy sectors (coal, oil, gas, refined oil and electricity). Electricity is generated from feedstock of either fossil fuels or non-fossil energy, assisted by a transformation and distribution sector. Global demand equals global supply for each good. In GRACE, production of primary energy is described by nested-CES functions. At the top level, energy output is a CES combination of a value-added intermediate aggregate and natural resources. At the second level, the value-added intermediate aggregate is a Leontief combination of intermediates and the value-added aggregate (a CES combination of capital and labor). 

As the functional forms of the demand are different in the two models (CES in GRACE; Cobb Douglas in FRISBEE), it is difficult to directly compare the modelling output. However, we do not harmonize the functional forms as this can strengthen the analyses. We emphasize that, other things being equal, substitution elasticities closer to one in GRACE reduce the difference between the two models (as the substitution elasticities between the various energy goods are all equal to one in FRISBEE). 

### 3.2. Reference Scenarios

The IEA’s Stated Policies Scenario (STEPS) stands out as a reference scenario for energy markets, energy security, and emissions and explores the implications of announced climate targets as well as existing energy policies [22]. In STEPS, primary energy demand grows by a quarter to 2040. Hence, there is no peak in global energy-related CO_2_ emissions until 2040, as the effects of an expanding economy and population on energy demand outweigh the need for a more efficient and lower-emissions energy system. By soft-linking GRACE and FRISBEE, the reference scenario simulated by each model aligns with the regional energy development of STEPS in [22], but we do not strive for a perfect match. STEPS ends in 2040, but we simulate our models to 2050 as we will compare policy scenarios with NZE IEA up to 2050.

Both models assume regional GDP growth rates, population development, and CO_2_ prices (and, to some extent, other policy-related variables) as in STEPS in [22]. Due to lack of data, we performed various estimations and calculations regarding regional CO_2_ prices and GDP growth rates, where pricing of CO_2_ emissions is by emissions trading systems or taxes although GRACE and FRISBEE only cover CO_2_ (see [17,23]). If the simulated demand (or supply) of the various energy goods in different regions is far off the levels in STEPS, we adjust central parameter values.

**Figure A1:**
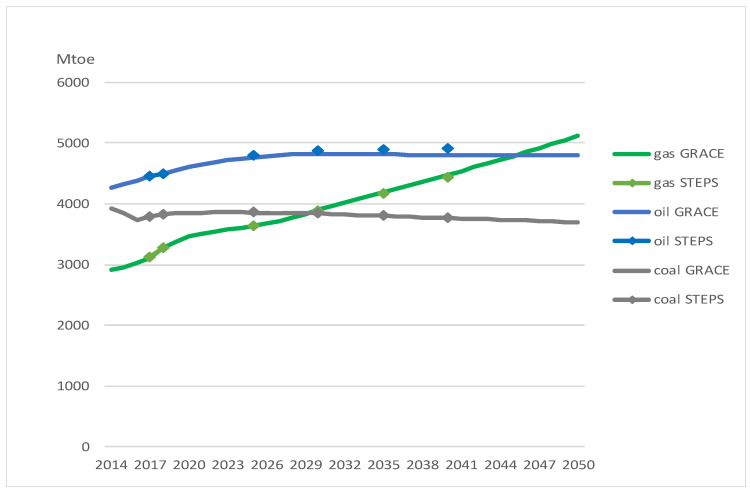
Comparison of fossil fuel consumption in STEPS and the reference scenario in GRACE.

**Figure A2:**
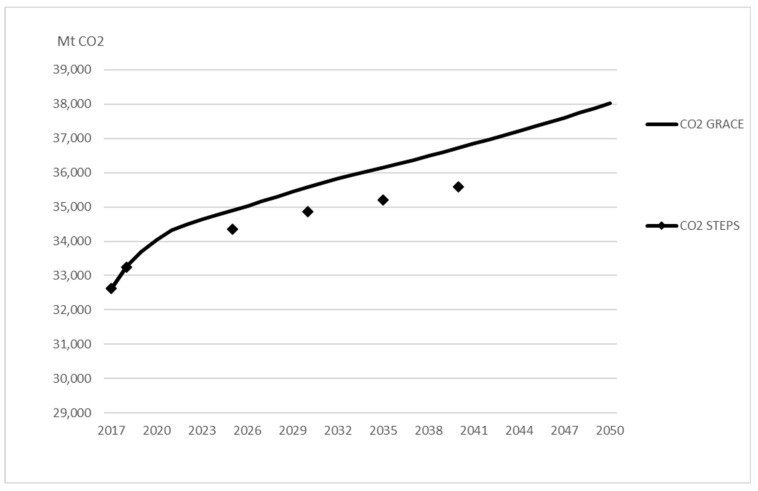
Comparison of CO_2_ emissions from fossil fuels in STEPS and the reference scenario in GRACE.

In the reference scenario simulated by GRACE, population development is set exogenously as in STEPS. The GDP path of STEPS is obtained through adjustment of factor-augmented technological changes with respect to capital and labor. Fossil fuel consumption of STEPS is approximated by adjustment of the endowment of reserves (or natural resources) available for production in these sectors and fossil fuel tax/subsidy rates for final users besides efficiency improvement of energy for final use. CO_2_ emissions from fossil fuels are then calculated by fossil fuel use multiplied with given emission factors (i.e., CO_2_ emissions per unit of fossil fuel use by sector and region). Hence, the population and GDP growth in the reference scenario of GRACE are the same as in STEPS. Figure A1 and Figure A2 in Appendix A show that consumption of fossil fuels and emissions of CO_2_ in the reference scenario of GRACE align quite well with STEPS.

**Figure A3:**
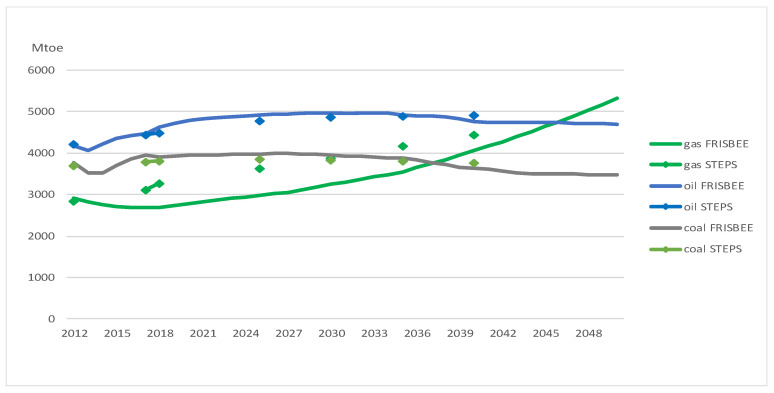
Comparison of primary fossil fuel consumption in STEPS and the reference scenario in FRISBEE.

**Figure A4:**
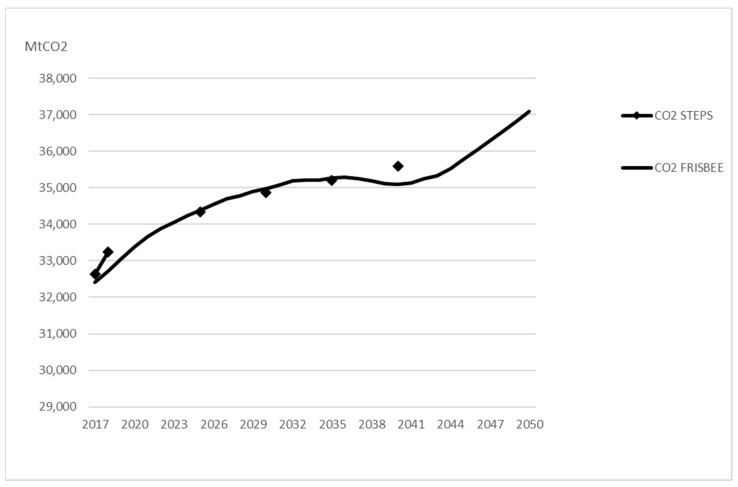
Comparison of CO_2_ emissions from fossil fuels in STEPS and the reference scenario in FRISBEE.

In FRISBEE, assumptions on population development, GDP growth rates, and CO_2_ prices in each region are taken from Cappelen et al. [4], which is also based on STEPS in IEA [22]. While renewable energy (incl. nuclear) in the power sector is endogenous in GRACE, it is exogenous in FRISBEE. We include the regional volumes of non-fossils in the power sector from STEPS in FRISBEE. Then, if the simulated demand (or supply) of the various energy goods in the different regions are far off targets in STEPS, we mainly adjust the income elasticities and the parameters for Autonomous Energy Efficiency Improvement (AEEI). For oil, we, in addition, include the exogenous oil price taken from IEA [22]. Figure A3 and Figure A4 in Appendix A show that consumption of fossil fuels and emissions of CO_2_ in the reference scenario of FRISBEE also align quite well with STEPS.

### 3.3. NZE Scenario in IEA

The NZE IEA scenario seems stricter than the scenarios in the IPCC special report, in terms of reaching net zero of total CO_2_ emissions in 2050. The CO_2_ emissions include emissions from burning fossil fuels and non-renewable wastes and emissions from fuel transformation and industrial processes, with the subtraction of CO_2_ removals from carbon capture, utilization, and storage (CCS). Focusing on the burning of fossil fuels, we study the effects on energy markets in the two models to roughly follow the pathway of total CO_2_ emissions in NZE IEA.

This study does not simulate removals of emissions for simplicity. Instead, we assume an exogenous pathway of CCS to remove CO_2_ over time, following IEA (2021), allowing for more emissions from fossil fuels in the two models equivalent to the exogenous amount of CO_2_ removals, at the same level as in NZE IEA. Although a given path of greenhouse gas (GHG hereafter) emissions can imply different temperature changes depending on which climate model is used, the 1.5 °C scenario in this study is defined as roughly following the pathway of total CO_2_ emissions from fossil fuels in NZE IEA. A discussion of which measures to use in GRACE and FRISBEE to make the scenarios align with NZE IEA can be found in Cappelen et al. [24].

### 3.4. The NZE Scenario in GRACE

We introduce six types of measures for all regions to reduce CO_2_ emissions (Table 1). All measures other than CO_2_ taxes are introduced from 2020.

Based on information available from [4], CO_2_ taxes by year and region were derived from NZE IEA [24]. We introduce the CO_2_ taxes as differences between the CO_2_ prices in NZE and STEPS, since our reference scenario simulated by GRACE does not introduce explicit CO_2_ prices.

In NZE IEA, much more renewable energy is used to generate electricity than in STEPS. The unit cost of renewable electricity is expected to decline over time. The assumed costs in NZE IEA are generally lower than in STEPS, in both absolute values and changes over time. A decline rate of 1 per cent of the costs means 1 per cent decline of all inputs to generate a given amount of electricity. In GRACE, this is interpreted as a 1 per cent improvement in Hicksian neutral technology, meaning if we keep the inputs of labor and capital constant, the generated electricity increases by 1 per cent.

Four technologies are modelled in GRACE to generate electricity fueled by coal, gas, oil and non-fossils. We assume that the cost for non-fossil-fueled electricity generation declines yearly by 3.0 per cent during 2020–2030, 1.5 per cent during 2030–2040, and 0.5 per cent after 2040, further than in the reference scenario, corresponding to a yearly rate of 1.7 per cent over the whole period of 2020–2050.

In NZE IEA, no electricity is assumed to be generated from unabated fossil fuels in 2050 and only 2 per cent of electricity generation is from fossil fuels with CCS. As GRACE has not included CCS so far, we introduce a modest restriction on electricity generated from fossil fuels either with or without CCS, i.e., we set upper limits for thermal electricity generation. Electricity generated from fossil fuels is assumed to be less than the previous-year level from 2030.

In NZE IEA, the renewable energy (either electricity or other types) is assumed to account for most of the final use in 2050. To achieve this, one must invest massively in infrastructure to facilitate the final energy users in replacing fossil fuels with renewable energy, which implies that considerable barriers to use renewable energy will gradually be removed. This makes it easier for the final users to substitute fossil fuels with renewable energy.

It has been argued that a high elasticity of substitution between clean and dirty inputs in an economy is crucial for sustainable and green growth [25]. In the specifications of nested CES production functions, [26] find that the substitution elasticity between clean and dirty energy inputs can significantly exceed one (around 2 in the electricity generation sector and 3 in the non-energy sectors), contrary to the findings in earlier interfuel substitution literature.

Hence, in GRACE, we allow for gradually larger substitution possibilities between fossil fuels and renewables by changing two parameters. Substitution elasticities between electricity and fossil fuels gradually become higher for final users (including producers and consumers) from 2020 to 2050, and so does the substitution elasticity between electricity generated from fossil fuels and renewable energy sources. For final users, the values of the substitution elasticities are assumed to increase from 0.5 (or 0.4) for producers (or households) in 2020 by 6.5 per cent yearly until 2040 and then the yearly rate is gradually lowered to 3.25 per cent in 2050, when the elasticities become 2.8 for producers and 2.2 for households. For the substitution elasticities between thermal and other types of power generation (1.386 for base load and 0.472 for peak load in 2020), the rates are 5.5 per cent until 2040 and are gradually lowered later until reaching 2.75 per cent in 2050. In 2050, the substitution elasticities become 6.0 for base load and 2.0 for peak load.

In GRACE, energy efficiency is implemented directly as a change over time in the parameter of energy-augmented technology in production functions and household consumption functions. Hence, it differs from the widely used energy intensity (energy use per unit GDP). If such a change is a 1 per cent increase, then it is interpreted as a 1 per cent decrease in energy input (or energy use) to produce a given amount of output (or welfare level), while all other inputs remain constant.

In NZE IEA, the energy intensity is reduced yearly by 4.2 per cent from 2020 to 2030 and 2.7 per cent from 2030 to 2050, compared to a decrease of 2.3 per cent in STEPS. Following the pattern of the energy intensity over time in the NZE IEA scenario, we assume that the parameter of energy-augmented technology for all final energy users of all regions in NZE GRACE increases by 1.5 per cent yearly until 2030 and 1.0 per cent between 2030 and 2050 compared to the level in STEPS. As a result, to produce a given amount of output, the assumed technology changes mean that the energy needed in 2050 is only 35 per cent of the energy needed in 2020 in NZE GRACE, other things being equal.

In NZE IEA, it is suggested to stop establishing new coal mines and mine extensions, and in addition to stop approving new oil and gas fields for development from 2021. This implies that the fossil reservoirs available for production are gradually reduced over time. Hence, this is interpreted as the gradual reduction of natural resources for fossil fuel production. In GRACE, we assume that the natural resources for fossil fuel production are gradually reduced from 2020 to become only 10 per cent for oil and 5.5 per cent for coal and gas of the 2050 level in the reference scenario. This measure alone pushes up the cost and price of fossil fuel production, resulting in less supply and demand.

### 3.5. The NZE Scenario in FRISBEE

We apply the following strategy for all three fossil fuels to establish the NZE scenario in FRISBEE. First, we implement the regional endogenous GDP growth rates from NZE GRACE discussed in the last section. We introduce the CO_2_ taxes by year and region derived from the NZE IEA scenario [24]. Further, we introduce exogenous amounts of non-fossils (renewables and nuclear) in the power sector. We have the regional volume of non-fossils per year and region for the Sustainable Development Scenario (SDS) in IEA [27]. We also have total global volume of non-fossils in NZE per year. We adjust the regional figures in SDS upwards with global non-fossil NZE/global non-fossils SDS. This makes electricity demand in FRISBEE align relatively close to the large increase in electricity demand in NZE IEA. In addition, we increase the efficiency improvement in demand for fossil fuels by 4 per cent to roughly align with IEA [4]. They apply a reduction in energy intensity (energy use/GDP) of more than 4 per cent per year between 2020 and 2030 and almost 3 per cent in the 2030-2050 period. Further, we apply the endogenous oil price from NZE GRACE. We also run model simulations with a halt in new investments in gas reserves and also reduce coal supply from 2021, which are key milestones in NZE IEA. Lastly, we consider if a further increase in energy efficiency is necessary to reduce demand even more. Hence, the measures we take into consideration are CO_2_ taxes (the same as that introduced in GRACE), increased renewables in the power sector (following NZE IEA), increased AEEI by 4 per cent for oil consumption and 8 per cent for coal and gas, the endogenous global oil price from GRACE, a stop in gas investments, and a lower supply for coal.

## 4. Results

### 4.1. The Energy Markets in the NZE Scenario in GRACE

**Figure 1:**
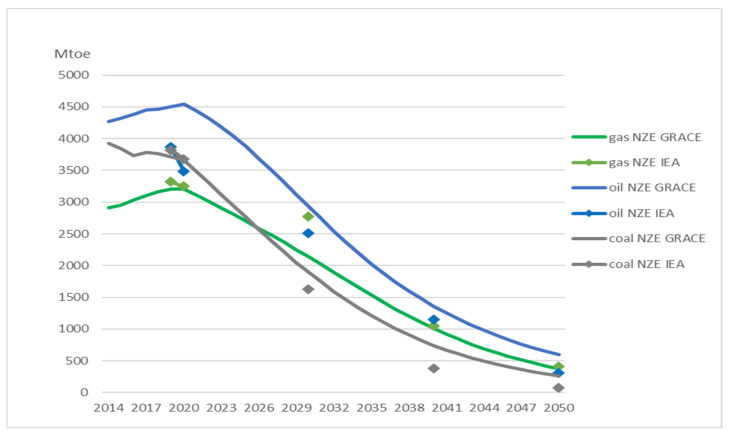
Comparison of primary fossil fuel consumption predicted in NZE IEA and NZE GRACE. Markers show the predicted values for the years available from IEA [4].

Figure 1 shows that oil demand in GRACE follows closely to the demand in NZE IEA but is somewhat higher over the simulated period, ranging from around 200 to 400 Mtoe. For the IEA, we only have data for specific years. In 2050, oil demand is reduced by 88 per cent from the reference scenario, lower than the reduction of 94 per cent in NZE IEA from STEPS (see Table 2). Gas demand in GRACE is always lower than in NZE IEA, above all in 2030 when it is 22 per cent below the NZE IEA target. Gas demand in GRACE ends up 93 per cent lower than the level in the reference scenario in 2050, somewhat lower than the 95 per cent in NZE IEA. Coal demand in GRACE is almost the same as NZE IEA in 2020 but is higher in the subsequent years. In 2050, coal demand is 93 per cent lower than in the reference scenario. This is lower than the reduction of 98 per cent in NZE IEA from STEPS (see Table 2).

**Figure 2:**
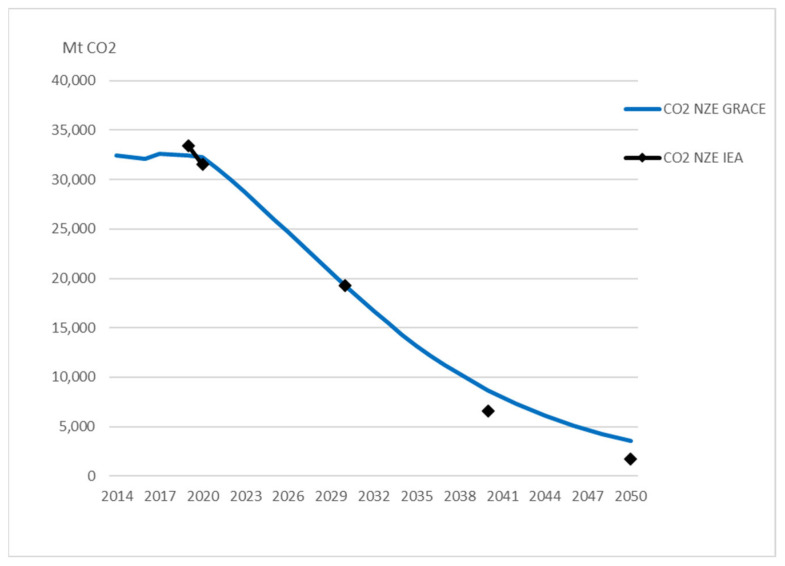
Comparison of CO_2_ emissions predicted in NZE IEA and NZE GRACE. Markers show the predicted values for the years available from IEA [4].

The relatively higher demand for coal and oil in GRACE leads to higher CO_2_ emissions from fossil fuels than in NZE IEA (Figure 2). In 2030, emissions are almost the same as in NZE IEA due to lower gas demand offsetting the effect of higher coal and oil demand. In 2050, emissions are 91 per cent below the reference scenario. This is lower than the reduction of 95 per cent in NZE IEA (see Table 2).

Table 3 shows the effect of each measure on oil, gas, and coal demand. Introducing CO_2_ taxes reduces oil demand only by 2 per cent in 2050 as oil can hardly be substituted by other energy goods at this level of substitution possibilities, not even for electricity in transport. The effect of the two measures directed towards power generation contributes only marginally to the reduction in oil demand, which is due to limited substitution possibilities, assumed as oil is only to a small extent used in power generation on a global scale. With increased substitution possibilities between oil and electricity, oil demand becomes 14 per cent lower than in the reference scenario. With improved efficiency of energy used by final users, oil demand is further reduced to become 21 per cent lower than in the reference scenario. When we assume reduced natural resources for oil supply, oil demand declines further to 88 per cent below the reference level in 2050.

Surprisingly, introducing CO_2_ taxes has a much larger effect on gas demand than oil and reduces gas demand by 25 per cent in 2050, mainly because of relatively higher carbon taxes per monetary unit (USD) for gas users in most regions. The carbon tax in NZE GRACE is the same for each ton of CO_2_ emissions in a region. As the carbon content of gas is lower, the carbon tax per ton gas is lower than that per ton coal. However, in the reference scenario simulated by GRACE, the gas price is already lower than the coal price in 2050 since the stated policies of governments encourage gas consumption rather than coal consumption. As a result, the carbon tax per USD gas becomes higher than that per USD coal. The two measures directed towards power generation further reduce gas demand by 5 percentage points. Easier substitution between electricity and fossil fuels for final users makes gas demand 56 per cent lower than in the reference scenario in 2050. Improved energy efficiency reduces gas demand further by only 4 percentage points from the reference level. Reduced natural resources for gas supply further reduces gas demand by 33 percentage points from the reference scenario.

Introducing CO_2_ taxes reduces coal demand by 10 per cent from the reference scenario, partly because coal is not easy to replace in the main coal-use regions such as, e.g., China, although coal is more carbonaceous than gas and is hit more by the CO_2_ tax. The joint effect of the two measures directed towards power generation reduces coal demand further by 5 percentage points. Increased substitution possibilities between electricity and fossil fuels leads to considerable reduction in coal demand by a further 43 percentage points from the level in the reference scenario. Improved energy efficiency contributes to reducing demand by 4 percentage points. When we, in addition, restrict natural resources availability for coal production, total demand in 2050 is reduced by 93 per cent from the reference level.

**Figure 3:**
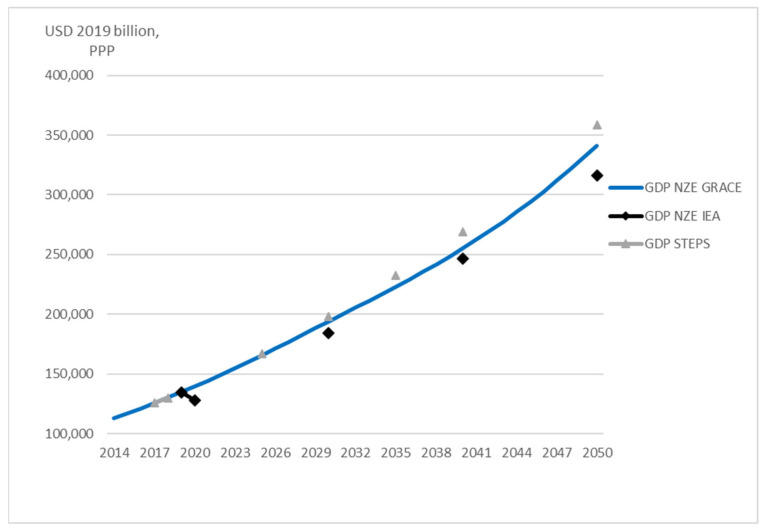
Comparison of world GDP predicted in STEPS, NZE IEA, and NZE GRACE. The 2050 GDP in STEPS is calculated from extrapolation for 2040-2050. Markers show the predicted values for the years available from IEA [4].

GDP in NZE GRACE is always higher than in NZE IEA from 2020 to 2050, and the difference slightly increases from 2040 (see Figure 3) due to, e.g., the ignorance in GRACE of the COVID-19 pandemic. This partially explains why the CO_2_ emissions in NZE GRACE are greater than in NZE IEA. However, GDP in NZE GRACE is lower than in the reference scenario (STEPS) and the difference increases over time. Notice that the model does not consider the cost of implementing the various measures and the simulated GDP might to a large extent underestimate the potential GDP losses to achieve the 1.5 °C scenario. In the measures we introduced, only CO_2_ taxes and reduced fossil fuel resources lead to GDP losses, while we do not consider the costs of the extensive use of renewable energy and the high pace of energy efficiency improvement.

**Figure 4:**
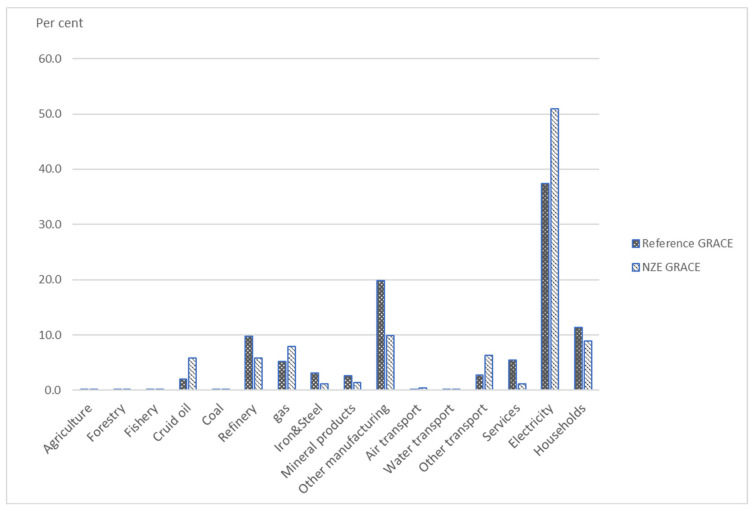
Sectoral shares of global gas demand in 2050 predicted by GRACE.

Gas is the least carbonaceous fossil fuel compared to coal and oil. Figure 4 shows the sectoral shares of global gas demand in 2050 in the reference scenario and NZE GRACE. The shares of gas demand increase in four sectors, power generation, other transport than air and water, gas production, and crude oil production, compared to the shares in the reference scenario. The reason is that it is relatively harder to replace gas with renewables in these sectors.

### 4.2. The Energy Markets in the NZE Scenario in FRISBEE

**Figure 5:**
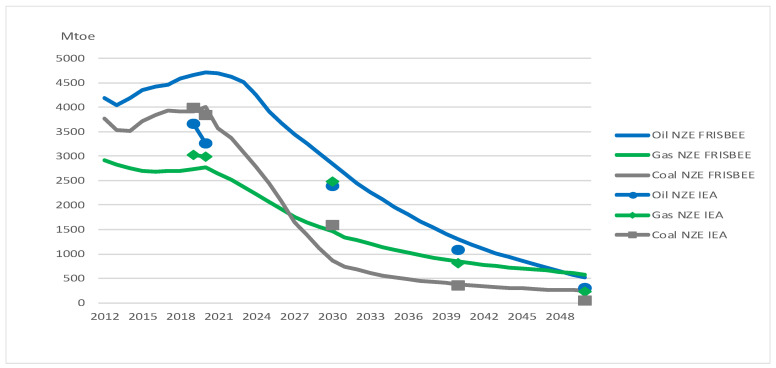
Comparison of primary fossil fuel consumption predicted in NZE IEA and NZE FRISBEE. Markers show the predicted values for the years available from IEA [4].

Figure 5 shows the aggregated effects on energy markets. Oil demand in FRISBEE is somewhat higher than demand in NZE IEA until 2040 (note that we do not consider short-term effects such as the decline in oil demand in 2020 that was due to the COVID-19 pandemic). In 2050, oil demand is reduced by 89 per cent from the reference scenario, almost at the same level as NZE GRACE and somewhat lower than the reduction of 94 per cent in NZE IEA from STEPS (see Table 2). We emphasize that the differences between our model results and NZE IEA in 2050 also are shown in Figure 1 and Figure 5.

Gas demand in FRISBEE is lower than NZE IEA in 2030, but at the same level in 2040. Gas demand in FRISBEE ends up 89 per cent lower than the level in the reference scenario in 2050, somewhat lower than the reduction of 93 per cent and 95 per cent in NZE GRACE and NZE IEA, respectively (see Table 2). The gas supply in FRISBEE is affected by, e.g., prior investments (which again is a function of the extensive reserve database). Hence, it is difficult to further adjust demand-side parameters to reduce demand even more (as it must equal supply).

Coal demand in FRISBEE is also lower than in NZE IEA in 2030, but at the same level in 2040. Further, in 2050, coal demand is 93 per cent lower than in the reference scenario. This is at the same level as in NZE GRACE and somewhat lower than the reduction of 98 per cent in NZE IEA from STEPS (see Table 2).

**Figure 6:**
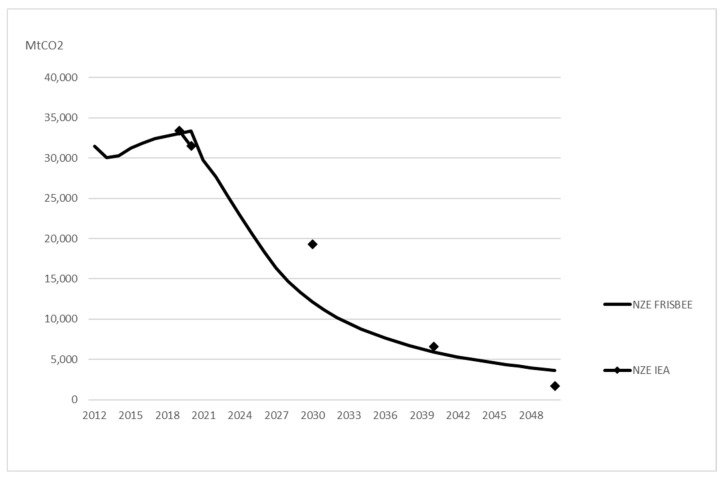
Comparison of CO_2_ emissions predicted in NZE IEA and NZE FRISBEE. Markers show the predicted values for the years available from IEA [4].

The effects on fossil fuels in FRISBEE lead to a decline in CO_2_ emissions, as shown in Figure 6. In 2030, emissions are lower than in NZE IEA due to lower coal and gas demand (even if oil demand is somewhat higher), but at the same level a decade later. In 2050, emissions are 91 per cent lower than in the reference scenario. This is at the same level as NZE GRACE and somewhat lower than the reduction of 95 per cent in NZE IEA (see Table 2).

Table 4 shows the effects of each measure on oil, gas, and coal demand in 2050. We emphasize that demand for all energy goods in either households or industry are log-linear functions of end-user prices, income, and AEEI, i.e., an increase in AEEI of *x* per cent leads to a decline in demand of *x* per cent for given prices and income.

Let us first take a closer look at oil demand. We see from Table 4 that introducing CO_2_ prices and increased renewables in power production reduces oil demand only by 14 per cent in 2050. The reason is primarily that oil is hardly used in power production on a global scale. With an AEEI of 4 per cent, which is roughly in line with improvements in energy intensity in NZE IEA and NZE GRACE, demand is further reduced. In addition, we insert the endogenous oil price from NZE GRACE. This oil price is around four times higher than the oil price in the reference scenario in FRISBEE, and leads to a reduction in oil demand by 89 per cent from the reference scenario.

We emphasize that stopping new oil investments from 2021 does not change oil demand. OPEC satisfies the residual between demand and non-OPEC supply at the exogenous oil price, that being the reference oil price in STEPS or the endogenous oil price in NZE GRACE. A credible defense of the price target requires surplus capacity. In our model, we therefore assume that OPEC will always invest sufficiently to maintain a capacity surplus of 10 per cent. As demand and non-OPEC supply are determined independently of each other, stopping non-OPEC investment in new reserves has no effect on total supply (=demand) (In NZE FRISBEE, OPEC manages to keep the required surplus capacity from 2021, when they increase production as non-OPEC supply declines).

**Figure 7:**
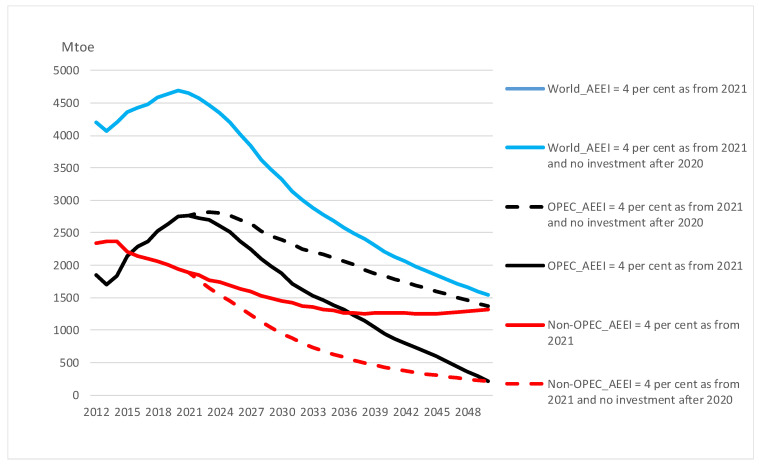
Oil supply; world, OPEC, and non-OPEC in various scenarios.

In the scenario with the endogenous oil price from NZE GRACE, OPEC is thrown out of the market if non-OPEC is allowed to continue to invest. Hence, we show the effect of stopping investment in the scenario with AEEI of 4 per cent and with the reference oil price. Figure 7 shows that the world oil supply declines by almost 70 per cent from 2021 to 2050. This is mainly due to a relatively steep decline in OPEC production and, to some extent, a moderate decrease in non-OPEC supply. OPEC satisfies the residual demand between global supply (=demand) and non-OPEC production over the period. Stopping investment in non-OPEC countries leads to a large reduction in their supply. However, world supply does not change as OPEC increases production to keep demand at the prevailing oil price.

If we assume that OPEC, instead of increasing supply to keep production constant, stops investments in non-OPEC when AEEI is 4 per cent, this reduces oil supply and consumption by 90 per cent from the reference scenario in 2050. Thus, a more realistic energy efficiency improvement of 4 per cent and a reference oil price would then be sufficient to let oil demand be relatively close to the NZE IEA target. However, we implement the high endogenous oil price from NZE GRACE and demand is reduced by 89 per cent from the reference scenario.

**Figure 8:**
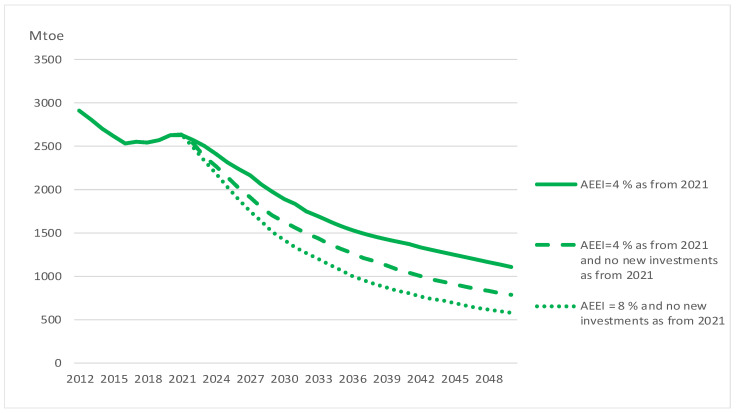
World gas supply in various scenarios.

Introducing CO_2_ prices and increased renewables in power production reduces gas demand by 33 per cent, mainly because gas is used in power production. By introducing a yearly AEEI of 4 per cent, gas demand is reduced to 80 per cent of the reference level in 2050. Stopping all new investments as from 2021 clearly also has an impact on gas demand as the reduction now is 85 per cent. By applying an AEEI of 8 per cent, demand declines further to 89 per cent of the reference scenario in 2050. We might add that stopping investment before introducing the AEEI of 4 per cent would make this measure stand out as relatively more important than the impression one gets from Table 4. Figure 8 shows world gas supply in various scenarios. When we move from a situation with an AEEI of 4 per cent to a situation where we, in addition, stop new investments, supply is only reduced by around one-third in 2050. We see that, if we, in addition, increase energy efficiency improvements, there are still enough profitable proven reserves today to sustain production until 2050 at around one-fifth of today’s level. Production is increasingly concentrated in resource-rich countries due to the large size and slow decline rates of their existing fields. In 2050, a large part of global gas is produced in the Middle East and Russia.

Introducing CO_2_ taxes and increased renewables in power production has a much larger effect on coal than on gas, as coal demand declines by 53 per cent from the reference scenario. Coal is also used in power production, but the main reason is that coal, in addition, is more carbonaceous than gas and thus is hit more by the CO_2_ tax. By introducing a yearly AEEI of 4 per cent, coal demand declines by 77 per cent from the reference level. By suppressing coal supply further, demand is 84 per cent lower in 2050. By applying an AEEI of 8 per cent, coal demand declines further to 93 per cent from the reference scenario in 2050.

## 5. Discussion

According to [4], technology alone is not enough to reach NZE in 2050. The active support of people is crucial. The report does not describe this as behavioral changes, but a mixture of low carbon technologies and people’s engagement, such as “buying an electric vehicle or insulating a loft”. However, it is emphasized that behavioral changes—meaning adjustments in everyday life that reduce, e.g., excessive energy consumption—are also needed. IEA [4] argues that this is especially important in richer parts of the world where energy-intensive lifestyles are the norm. Behavioral changes include cycling or walking instead of driving, turning down heating, and going on holiday nearer to home. Total accumulated CO_2_ emissions in the NZE between 2021 and 2050 are around 4 per cent less than they would be without such behavioral changes.

Neither GRACE nor FRISBEE include behavioral changes or changes in preferences. Behind demand and supply lies traditional maximization of utility and profit. However, if we include behavioral changes to the extent that the IEA does, reduction in emissions in 2050 in both models might increase closer to the level of NZE IEA, as Table 2 shows. Further, we included both energy use with CCS and unabated use in our simulations when making the comparison in Table 2. Hence, if CCS were included in our models, reduction in energy use and emissions from the reference scenario could be closer to 100 per cent. Further, opposed to the IEA, we did not include hydrogen in our scenarios.

Even with the introduction of CCS and energy alternatives such as hydrogen, strict measures are necessary to reduce emissions to achieve the demand required by a 1.5 °C scenario. A major worldwide push to increase energy efficiency is an essential part of the efforts to reduce energy use and emissions. We introduced an efficiency improvement of 8 per cent per year in FRISBEE for gas and coal, but we see from Table 2 and the discussion related to the table that large reductions in emissions can be obtained by an AEEI of 4 per cent. For oil, an AEEI of 4 per cent is enough. Both NZE GRACE and NZE IEA operate with reductions in energy intensity. Both apply a reduction in energy intensity (energy use/GDP) of around more than 4 per cent per year between 2020 and 2030 and almost 3 per cent in the 2030–2050 period. According to IEA [4], 4 per cent is about three times the average intensity decline rate achieved over the last two decades. To accomplish such an increase would, of course, be a very challenging task.

The IEA has been criticized for applying exogenous growth in GDP in their scenarios. Mohn [28] emphasizes that empirical models of energy economics and climate change should open for the indigenization of economic activity. For the IEA, this could allow for variation in energy prices and policies to imply corresponding variation in economic growth between the different scenarios. IEA [4] uses similar exogenous GDP growth in both NZE and the reference scenario. GRACE endogenizes the growth in NZE, which is inserted in NZE FRISBEE and leads to a somewhat lower growth compared to the STEPS scenario. However, we did not take into consideration all costs connected to the climate policies, e.g., efficiency improvement, and how these costs may affect future growth. Empirical estimates of such costs vary considerably and depend on sectoral and regional environment [11].

We apply the endogenous oil price from NZE GRACE in the NZE FRISBEE scenario. This oil price is four times higher than the reference oil price in STEPS, reaching over 400 USD (2012 prices) per barrel in 2050. Is this realistic? IMF [29] has shown that the NZE scenario can be consistent with both increasing and declining oil prices. When they only consider demand-side policies, oil prices could decline to 20 USD in 2030. When reductions in oil production are driven by supply-side measures, like the one we implement in NZE GRACE, this would result in a strong upward pressure, taking prices to roughly 190 USD a barrel in 2030. The latter oil price is somewhat higher than our price in 2030 in the NZE scenarios.

In the simulation of GRACE, the lower availability of natural resources in fossil production is crucial for reducing CO_2_ by pushing up the cost of fossil fuel production. On the other hand, we must introduce relatively high levels of efficiency improvement in FRISBEE for gas and coal while allowing extraction to phase out gradually over time to approach the emission reduction level of NZE IEA. As a result, the gas and coal prices are relatively low in FRISBEE and can potentially result in more gas and coal consumption, a well-known phenomenon of rebound effects [11,12]. As FRISBEE is a partial equilibrium model, the model does not consider the potential economy-wide rebound effects on energy consumption of energy efficiency improvement. However, the rebound effects are taken into account in the macroeconomic model GRACE and thus lead to more energy consumption, which has to be controlled by supply-side measures, e.g., the lower availability of natural resources in fossil production in our case.

When we introduce CO_2_ taxes, increased use of renewables, and an efficiency improvement of around 4 per cent, emissions are reduced more in FRISBEE than in GRACE in 2050. In NZE GRACE, lower availability of oil resources, but also to some extent gas and coal resources, are paramount to reach the desired emission targets. In NZE FRISBEE, it is to some extent important that gas investment in new reserves is stopped from 2021 and that coal supply is reduced. Oil investments decline rapidly due to the high oil price implemented from NZE GRACE. One might question if we can see signs of a halt in development of new oil, natural gas, and coal reserves or a reduction in investments. IEA [30] concludes that, even if the pace of growth in renewable investment has accelerated since 2020, today’s fossil fuel spending is too high for a pathway aligned with limiting global warming to 1.5 °C.

Another milestone in NZE IEA is in power generation, where overall net-zero-emissions electricity is achieved by 2040 globally. This requires no additional coal power stations, with generation from unabated plants phased out by 2040 globally. Unabated natural gas power generation must fall by 90 per cent globally by 2040 from 2020. Global Energy Monitor [31] shows that a large amount of new coal-fired power capacity is still being built. In addition, Wilson et al. [32] conclude that these milestones on the route to NZE are at odds with the current trajectory of our energy system, with significant expansion planned in both fossil fuel reserves and fossil fuel power. They are also at odds with the financial flows supporting this expansion, despite financial institutions with 130 trillion USD in assets committing at COP26 in Glasgow in 2021 to align their portfolios with net zero by 2050. For fossil fuel production, the case studies in Wilson et al. [32] show that oil and gas companies’ financing are continuing to expand fossil fuel reserves, allocating approximately 10 per cent of capital expenditures (CAPEX) to exploration-related activities.

## 6. Conclusions

A net zero pathway to 2050 in the energy sector was created in this study by soft-linking an energy model with a macroeconomic model. Based on a reference scenario assuming the same population growth, GDP growth rates, and CO_2_ prices, both models were modified to roughly follow the same pathway of both demand for fossil fuels and total CO_2_ emissions from the combustion of fossil fuels as in the NZE scenario from IEA [4]. We apply an endogenous GDP effect in our NZE scenarios, contrary to, e.g., NZE IEA where GDP growth is exogenous. To achieve our NZE scenarios, various mitigation measures were implemented simultaneously in our models, including CO_2_ taxes, improved energy efficiency, more renewables in electricity production and other sectors, easier substitution between electricity and fossil fuels for final users, and drastically limiting future production of fossil fuels.

We find that, to achieve the net zero pathway, the energy model needs relatively high levels of efficiency improvement and the macroeconomic model needs relatively strict supply-side measures to reduce production of fossil fuels. This is likely related to the potential role of economy-wide rebound effects on energy consumption of energy efficiency improvement. Such rebound effects are simulated by the macroeconomic model but cannot be taken into consideration by the partial equilibrium energy model.

We conclude that net zero is possible by introducing very strict measures in a modelling world. However, the rate of, e.g., energy efficiency improvement needed is far above what has been achieved in the past. Further, even if renewables are on the rise, the world does not seem to be on a trend of sufficiently declining fossil fuel use, as investment in both new reserves and new fossil power plants continues. This reminds us what is needed for a 1.5 °C world, although it will be very challenging, if not impossible, to make different governments agree on such measures.

## Figures and Tables

**Table 1:** The measures introduced in GRACE to achieve a net zero scenario.

Measure	Description
CO_2_ taxes	Regional CO_2_ taxes following NZE IEA are introduced [24]
Lower cost of power generation	Non-thermal electricity generation costs are reduced by 3.0, 1.5, and 0.5 per cent over the periods 2020–2030, 2030–2040, and 2040–2050, respectively.
Upper limit of thermal power	The generation of thermal power in a year after 2020 is not allowed to be more than in the previous year.
Increased substitution between fossils and power	Substitution elasticities between the use of fossil fuels and electricity are increased over time.
Improved energy efficiency	Energy-augmented technology for all final energy users increases by 1.5 per cent yearly in 2020–2030 and 1.0 per cent in 2030–2050 over the level in the reference scenario.
Reducing fossil reservoirs	The natural resources used in fossil fuel production in 2050 dramatically reduce to become only 10 per cent for oil and 5.5 per cent for coal and gas of the levels in the reference scenario.

**Table 2:** Reduction in predicted final demand and CO_2_ emissions from reference scenarios in 2050; per cent.

	NZE FRISBEE	NZE GRACE	NZE IEA *
Oil	89	88	94
Gas	89	93	95
Coal	93	93	98
CO_2_	91	91	95

* Calculated from extrapolation for 2040-50. We add the amount of fossil fuel use with CCS in NZE IEA to allow for additional emissions in the two models. With only unabated use of gas and coal, the emission reduction in NZE IEA would have been closer to 100 per cent, especially when it comes to coal.

**Table 3:** Reduction in predicted primary energy demand in 2050 due to each additional measure in NZE GRACE.

Measures Adopted in GRACE	2050					
	Oil		Gas		Coal	
Consumption in reference scenario	4797	mtoe	5114	mtoe	3699	mtoe
CO_2_ taxes	2%		25%		10%	
+ Cost changes in power generation	2%		25%		11%	
+ Upper limits on thermal power	2%		30%		15%	
+ Increased substitution between fossil fuels and renewables	14%		56%		62%	
+ Energy efficiency	21%		60%		66%	
+ Reduced natural resources for fossil fuel production	88%		93%		93%	

**Table 4:** Reduction in predicted primary energy demand in 2050 due to each measure in NZE FRISBEE.

	2050		
	Oil	Gas	Coal
Reference scenario	4703 mtoe	5318 mtoe	3475 mtoe
CO_2_ taxes and increased renewables in power sector	14%	33%	53%
+ AEEI = 4% (Includes increased bioenergy in households and industry.)	67%	80%	77%
+ Endogenous oil price	89%		
+ Stopping gas investments (Oil demand is not influenced by investments (as explained in the text).)		85%	
+ Lower supply for coal			84%
+ AEEI = 8%		89%	93%

## Data Availability

We declare that the data and materials presented in the article are reliable.

## Appendix A. More Details on Reference Scenarios

How does the reference scenario in GRACE compare with STEPS? Fossil fuel consumption and CO_2_ emissions are slightly different from STEPS (Figure A1) due to sectoral and regional differences between STEPS and the reference scenario of GRACE. We see from Figure A1 that oil demand is generally marginally lower in GRACE than in STEPS and, in 2040, demand is 2 per cent lower. Demand for coal and gas is almost the same as in STEPS. Figure A2 shows that CO_2_ emissions in GRACE are marginally higher than in STEPS—emissions are 3 per cent higher in 2040.

How does the FRISBEE reference scenario align with STEPS? We see from Figure A3 that oil demand generally is marginally higher in FRISBEE than in STEPS up to 2030, but is marginally lower in 2040. Demand for gas is generally lower than in STEPS and is almost 9 per cent below the level in STEPS in 2040 (A simple extrapolation of gas demand in STEPS after 2040 shows that demand in FRISBEE is 12 per cent higher in 2050). The gas price is endogenous in FRISBEE and supply is affected by, e.g., prior investments (which again is a function of the extensive reserve database). Hence, it is difficult to adjust demand-side parameters to change demand (as it equals supply). Coal demand is generally marginally higher than in STEPS up to 2030, but is slightly lower in 2040 (We also adjust demand for electricity and bioenergy to the final end-users households and industry). Figure A4 shows that CO_2_ emissions in FRISBEE ends up marginally lower than in STEPS in 2040.
